# Genome-wide analysis of *An. gambiae s.s.* reveal no genetic differences between indoor- and outdoor-biting populations in The Gambia

**DOI:** 10.1186/1475-2875-13-S1-P31

**Published:** 2014-09-22

**Authors:** Danica Fabrigar

**Affiliations:** 1 Department of Zoology, University of Oxford, Oxford, UK

## Background

Indoor-based control strategies against Anopheles vectors remain indispensable in the current effort to reduce malaria transmission. However recent reports indicate that vectors are increasingly biting outdoors in areas where coverage of insecticide-treated nets is high - potentially a sign of behavioural adaptation [1,2]. Using whole genome deep sequencing, we investigated whether there is a genetic difference between indoor and outdoor-biting populations of *An. gambiae s.s.*

## Materials and methods

*An. gambiae s.s.* were collected by human landing catches from indoor and outdoor locations approximately 60-200 m apart in The Gambia. Genomic analysis was performed on single-nucleotide polymorphisms (SNPs) ascertained from whole-genome sequencing.

We examined the diversity, divergence and deviation from neutrality of different genomic regions to look for evidence of genetic differentiation between the two populations. We also performed a genome-wide association study (GWAS) to test for any associations of SNPs with the outdoor-biting phenotype.

## Results

Estimates of population structure show weak differentiation between the two populations (F_st_ = 0.00004). There was no difference in the pattern of nucleotide diversity and Tajima’s D statistics between the two populations which exhibited variations within and between chromosomes. Linkage disequilibrium (LD) was low across all chromosomes, except for elevated peaks within inversions and centro-/telomeric regions. With no known loci implicated in biting-behaviour, we examined all loci and defined a cut-off at *P* < 1 x 10^–5^ for significant associations yielding a total of 53 SNPs of interest. We found that most of these SNPs were in non-coding regions.

## Conclusions

This is the first study investigating the genetic differentiation between indoor and outdoor-biting populations. We detected very little genetic differences between the two populations except for a few SNPs that were identified in the GWAS. These SNPs are currently under investigation in a comparison panel comprising of Ugandan samples.
